# Factors Associated with High Parent- and Youth-Rated Irritability Score in Early-Onset Mood Disorders: A Cross-Sectional Study with the Affective Reactivity Index (ARI)

**DOI:** 10.3390/brainsci14060611

**Published:** 2024-06-19

**Authors:** Giulia Serra, Massimo Apicella, Elisa Andracchio, Giorgia Della Santa, Caterina Lanza, Monia Trasolini, Maria Elena Iannoni, Gino Maglio, Stefano Vicari

**Affiliations:** 1 Child & Adolescent Neuropsychiatry Unit, Bambino Gesù Children’s Hospital, IRCCS, 00146 Rome, Italy; elisa.andracchio@opbg.net (E.A.); giorgia.dellasanta@opbg.net (G.D.S.); caterinalanza95@gmail.com (C.L.); monia.trasolini@gmail.com (M.T.); m.elena.iannoni@gmail.com (M.E.I.); gino.maglio@opbg.net (G.M.); stefano.vicari@opbg.net (S.V.); 2 Department of Neuroscience, Catholic University of the Sacred Heart, 00168 Rome, Italy; 3 Department of Life Science and Public Health, Catholic University of the Sacred Heart, 00168 Rome, Italy

**Keywords:** irritability, bipolar disorder, major depression, mixed, cycling, children, adolescent, affective reactivity index, mood dysregulation

## Abstract

Correct classification of irritability is extremely important to assess prognosis and treatment indications of juvenile mood disorders. We assessed factors associated with low versus high parent- and self-rated irritability using the affective reactivity index (ARI) in a sample of 289 adolescents diagnosed with a bipolar or a major depressive disorder. Bivariate analyses were followed by multilinear logistic regression model. Factors significantly and independently associated with high versus low parent-rated ARI score were: more severe emotional dysregulation and bipolar disorders diagnosis. Factors significantly and independently associated with high versus low self-rated ARI score were: lower children depression rating scale (CDRS-R) difficulty of having fun item score, greater children depression inventory (CDI-2) self-report score, more severe emotional dysregulation, and greater CDRS-R appetite disturbance item score. High parent-rated irritability was strictly related with a bipolar disorder diagnosis, whereas high youth-rated irritability was related to depressive phenotype characterized by appetite/food-intake dysregulation, mood lability, and less anhedonia and apathy.

## 1. Introduction

Irritability is one of the most common reasons for consulting a child and adolescent psychiatrist. Severe chronic irritability during childhood also represents an important risk factor for psychopathology during adolescence and during later adulthood, in particular for mood and anxiety disorders. Indeed, more than a third of children and adolescents diagnosed with a first major depressive episode (MDE) present an irritable mood and it is widely reported that juvenile MDEs progress to bipolar disorder (BD) during adulthood in 20 to 50% of cases [1]. For this reason, studies on irritability have increased significantly in recent decades, highlighting correlations with differential diagnoses and leading to unsolved controversies regarding the diagnosis of pediatric BD (PBD).

It is important, therefore, to have a clear conceptualization of irritability, in which we recognize a tonic component (irritable mood most of the time) and a phasic component (outbursts), which may be present to a different extent in specific clinical populations. This also allows us to distinguish irritability from anger and aggression, although there is a significant correlation between the three constructs.

Both tonic and phasic components of irritability are distinctive features of the diagnosis of disruptive mood dysregulation disorder (DMDD) of the fifth edition of the Diagnostic and Statistical Manual of Mental Disorders (DSM-5) [2], which describes a phenotype with severe chronic irritability, modeled on the conceptualization of severe mood dysregulation (SMD) by Liebenluft and colleagues [3]. On the other hand, irritability is included among the DSM-5 diagnostic criteria for several psychiatric disorders, including BD, oppositional defiant disorder (ODD), generalized anxiety disorder (GAD), post-traumatic stress disorder (PTSD), and major depressive episodes (MDEs), and therefore, it is considered a trans-diagnostic non-specific clinical feature.

Controversies on the diagnosis of PBD still exist. Some studies [4] have interpreted symptoms such as psychomotor agitation, crowded thoughts, and chronic irritability—often comorbid with anxiety disorders, attention deficit/hyperactivity disorder (ADHD), and conduct disorder—as a developmental subtype of BD, referring to them as “pediatric mania”. Liebenluft and colleagues, about 20 years ago, also described a “broad” phenotype of PBD [5], characterized by severe irritability and a chronic course, without distinct MDEs and hypomanic episodes. However, following longitudinal studies have clarified that this phenotype usually does not evolve into adult BD [6]. Therefore, DSM-5 introduced the diagnosis of DMDD in the chapter of depressive and related disorders, in order to limit overdiagnosis of PBD in chronically irritable children.

However, irritability has continued to be a discussed issue in child and adolescent psychiatry and a correct classification of this symptom can be difficult, but it can have important implications on prognosis and treatment.

Recent studies have highlighted that specific clinical descriptions of current mood symptoms, both depressive and hypomanic (even when subsyndromal), can differentiate unipolar from bipolar depression in adolescents. Based on this consideration, our aim is to see how a specific description of the symptom of irritability can characterize different phenotypes in major depressive and bipolar mood disorders in children and adolescents. For this purpose, we used the affective reactivity index (ARI), a scale developed by Stringaris and colleagues and also validated for the Italian population [7], which evaluates chronic irritability through a questionnaire administered to the subject and to their parents/guardians. The questionnaire asks to report irritability levels in the previous 6 months, and its score is related to SMD diagnosis [8]. No cutoffs are proposed for ARI in current literature. Mulraney et al., in an exploratory work [9], suggested a cutoff of 4 as the marker of significant psychopathology. Interestingly, this value is also coincident with the median and mean for both ARI modules in a wider and heterogeneous clinical population of outpatients, mostly with ADHD and anxiety disorders, but also with MDEs, at first assessment or in treatment [10].

There is evidence that irritability is a construct which characterizes a distinct subset of mood disorders presentations in adults [11,12,13,14,15] and in children [4,16,17,18], whose correct diagnostic categorization is challenging and nosologic classification is debated. However, from a dimensional perspective, chronic severe irritability is a construct with recognized stability [19] and distinctive neurobiological features [4,11,13,14,15,16,17,18]. We aim to study pediatric onset mood disorders with low versus high irritability at first clinical presentation using parent and youth modules of the affective reactivity index (PARI and YARI, respectively). Our clinical population is homogeneously characterized by adolescent outpatients with confirmed diagnosis of early onset unipolar or bipolar mood disorders. We explored distinctive features of high versus low irritability presentations caught by ARI and its correlation with affective episodes and with DSM-5 categorical diagnoses, also controlling for other psychopathological symptoms. Our primary hypothesis is that, among adolescents with severe young-onset mood disorders, quantifying irritability with ARI may help to identify a higher irritability phenotype with distinct psychopathological features, possibly helping differential diagnosis of early onset mood disorder at first presentation.

Then, we investigated psychopathological traits significantly associated with high versus low ARI scores in two multivariable logistic regression model (separately for PARI and YARI), in order to assess which ones help us to explain trait variability measured by ARI.

## 2. Materials and Methods

### 2.1. Participants

We performed a cross-sectional study on outpatients referred to the mood disorder program of Bambino Gesù Children’s Hospital in Rome, a III level referral children hospital in Italy, Rome. We collected data on consecutive patients from January 2017 to April 2022. The selection of patients has been based on their symptoms severity before being referred. Possible referring sources were primary care (pediatricians), secondary care (child psychiatrists in National Healthcare Centers), or emergency department.

Included subjects were children and adolescents aged younger than 18 years at evaluation with a confirmed diagnosis of Major Depressive Disorder (MDD), persistent depressive disorder (PDD) with current intermittent MDE, bipolar disorder type I (BD-I), II (BD-II), or not otherwise specified (NOS), currently experiencing a major depressive or (hypo)manic episode, of any level of severity. Categorical diagnoses were made by clinicians and confirmed with the Kiddie-Schedule for Affective Disorders and Schizophrenia for School-aged Children, Present and Lifetime version (K-SADS-PL) [20] following DSM-5 criteria. Two authors (G.S. and G.M.) reviewed the clinical charts independently, and asked for the agreement of a third author (M.A.) to solve some minor discrepancies, and patients were included only after reaching agreement on the diagnosis.

We excluded patients with diagnosis of intellectual disability and/or autism spectrum disorder and patients with a diagnosis of substance induced mood disorder and/or mood disorder due to another medical condition according to DSM-5. Parents/legal guardians provided written informed consent for anonymous reports of data in aggregate form, in compliance with research ethics. Data collected were stored in individual clinical records. Considering the retrospective nature of the analysis, the current study did not require the approval of the local ethics committee according to current legislation, but a notification was sent. Data were retrospectively analyzed in line with personal data protection policies.

### 2.2. Assessment

All participants underwent a psychopathological assessment during three visits for 9 h of assessment per patient. Parents/caregivers were always interviewed as informative sources on patient’s psychopathology. Assessment has been fully described in previous studies [21,22]

We used the following assessment tools:

- Kiddie-Schedule for Affective Disorders and Schizophrenia for School-aged Children, Present and Lifetime version (K SADS-PL) [20] has been used to assess current and past (lifetime) psychopathological features and psychiatric disorders according to DSM-5 criteria [2]. DSM-5 disorder diagnosis is formulated by the clinician after interviewing patients and their parents/caregivers. Mixed features of current depressive or (hypo)manic episodes were defined as in DSM-5. “Cycling” mood features have been described as in Judd et al. 2002 [23] and Birmaher et al. 2009 [24], on the wake of DSM-IV definitions, as a combination of ultra-rapid mood switches between manic and depressive symptoms within hours or few days during current affective episodes, a state characterized by rapid significant changes in mood polarity, which is not equivalent of DSM-5 rapid cycling specifier.
- Affective Reactivity Index (ARI) has been used to measure irritability. This scale includes two self-rating modules, one for the young patient (YARI) and one for parents/caregivers (PARI). Each module is made of six items which investigate the occurrence of irritable feelings and behaviors in the previous six months. A seventh item asks to rate the level of impairment due to irritability. Each item has a three-level response category, i.e., ‘not true’, ‘somewhat true’, and ‘certainly true’, scored as 0, 1 or 2, respectively, giving a total range of possible scores from 0 to 12. ARI is a reliable tool, with good internal consistency (Cronbach’s α = 0.87) [7] and it has been validated for Italian population [7]. The first six items represent a single factor and correlate significantly with the level of impairment rated in the seventh item. We analyzed separately the total score of YARI and the total score of PARI, by summing the scores of the first six items in each module. The impairment item score in YARI and PARI has also been analyzed separately.
- Children’s Depression Rating Scale–Revisited (CDRS-R) is a semi-structured interview, made of 17 items, administered to patients and to parents/caregivers [25] to assess the severity of a current depressive episode. It is a clinician rated instrument which requires the interviewer to provide a score for each of 18 items after completing the interviews with patients and caregivers on symptoms during the days prior to the interview.

Total score, ranging between 18 and 120, is provided by summing the single items scores. Total scores of 30 or higher are considered compatible with active depression; total scores of 55 or higher are considered indicative of severe depression.

At first, we analyzed raw total score and single items scores (school dysfunctions; difficulties in having fun; difficulties in interpersonal relationships; sleeping disorders; appetite disorders; excessive fatigue; psychosomatic complaints; irritability; excessive guilt; low self-esteem; depressive feelings; morbid ideas; suicidal ideation; excessive crying; reduced facial expressions; slow speech, and motor hypoactivity). In a second moment, we subtracted the irritability item score from the total score and we analyzed correlations between it and the different levels of irritability rated by ARI scores.

- K-SADS Mania Rating Scale (KMRS) [20] is a semi-structured interview, made of 14 items (Elated or expansive mood, Irritable mood, Grandiosity, Decreased need for sleep, pressured speech, Racing thoughts, Distractibility, increased goal-directed activity, motor hyperactivity, Poor judgment, Unusual energy, Hallucinations, Delusions, Mood lability), administered to patients. It is a clinician rated instrument, focusing on manic symptoms during the worst week of the past month. Each item is rated on a 6-point scale, except for distractibility, which is rated on a 5-point scale. Total score, ranging from 1 to 68, is calculated by summing the scores of each item, minus 13. Scores of 12 or higher are considered indicative of clinically significant (hypo)manic symptoms.

At first, we analyzed raw total score and single items score (euphoria, irritability and anger, mood lability, reduced need for sleep, crowded thoughts, increased energy, increased activities, motor hyperactivity, grandiosity, rapid or pressured speech, distractibility, impaired judgment, and hallucinations and delusions). In a second moment, we subtracted the irritability item score from the total score and we analyzed correlations between it and the different levels of irritability rated by ARI score.

- Clinical Global Assessment Scale (C-GAS) is used to score functional impairment during past two weeks [26]. On a scale ranging from 0 to 100, lower scores indicate lower levels of general functioning. It is a clinician-rated instrument.
- Child Behavior Checklist for ages 6–18 years (CBCL) [27] is extensively used to assess cross-cutting symptoms related to different psychopathological conditions in the actual state and during past 6 months. It provides scores for specific syndromal scales (withdrawn-depressed, somatic complaints, anxious-depression, social problems, thought problems, attention problems, rule-breaking behavior, and aggressive behavior). By combining the severity of the different symptoms, three behavior rating scales—internalizing symptoms, externalizing symptoms, and total behavioral problems—can be calculated. Subitems of these three scales (attention problems, aggression, and anxious-depressed syndromal scales) can be summed to estimate an “AAA” CBCL index, indicative of Deficient Emotional Self-Regulation (DESR), at scores between 180 and 210 (standard deviations, SD of 1–2), and as meeting criteria for a Severe DESR at a score of >210 (>2 SD) [28]. CBCL has been administered to parents/caregivers.
- Children Depression Inventory-2 (CDI-2) [29], one of the most used tools to self/parent-reported depressive symptoms during past two weeks in children. It has been administered since 2018, after availability in the Italian version, to patients and, separately, to parents/caregivers. The full form of the patient report has 28 items with a response scale from 0 to 2, whereas the parent module has 17 items with a response scale from 0 to 3. Composite total score is given by the sum of the items has been converted in t score. A t score < 60 indicates low depression symptoms, a t score between 60 and 64 indicates presence of mild depression symptoms, a t score between 65 and 69 indicates high level of depression symptoms, and a t score of 70 or more indicates severe depression symptoms. We analyzed the t scores.
- Multidimensional Anxiety Scale for Children-2 (MASC-2) [30], one of the most used tools to self-report anxiety symptoms in children during past weeks, has been administered since 2018, after availability in the Italian version, to patients and, separately, to parents/caregivers. Both patient and parent report module have 50 items with a response scale from 0 to 3. Composite total score is given by the sum of the items, which is then converted in a t score. A t score < 55 indicates low anxiety symptoms, a t score between 56 and 59 indicates presence of high/average anxiety symptoms, a t score between 60 and 64 indicates presence of mildly elevated anxiety symptoms, a t score between 65 and 69 indicates high level of anxiety symptoms, and a t score of 70 or more indicates severe anxiety symptoms. We analyzed the t scores.

Suicidal ideation and attempts were defined as in Posner (2011). Non-suicidal self-injury (NSSI) was defined as in DSM-5.

### 2.3. Statistical Analysis

Categorical variables were presented as number of observations and frequency rate. Continuous variables were presented as mean and SD.

The median PARI score in our population was 4, and significantly lower than YARI (YARI median = 6 vs. PARI median = 4; *p* < 0.001) and inter-rater reliability for ARI between patients and parents was less than optimal (Inter-Class Correlation coefficient = 0.4; 95%IC 0.29 to 0.49; F = 2.31, *p* > 0.001). For these reasons, we chose a different empiric cutoff for YARI and PARI modules. This is in line with recent literature recommendation not to average PARI and YARI scores [31].

Patients were in this way dichotomized for further analyses into high vs. low self/parent-rated irritability groups according to the cutoffs of 4 for PARI and 6 for YARI. Associations between unpaired categorical variables had been tested with Pearson’s chi-square test. Fisher’s exact test was used in case more than 20% of cells in the contingency table had expected frequencies < 5 [32]. Mean difference of continuous variables between two groups has been tested with Student’s t-test for unpaired data.

We performed two logistic regression models, respectively, for PARI and for YARI, in order to asses which of the studied variables significantly correlates with the high parent/youth-rated irritability phenotype.

In each model, belonging to high parent/youth ARI score was set as a predicted variable, whereas diagnostic and psychopathological variables found significantly different at previous bivariate analyses were tested as predictors.

Non-significant variables in each model were removed with a stepwise backward manner to improve model fit. Odds ratios, with their 95% confidence intervals (95% CI), *p*-values, and Wald test statistics are presented. Intercept has been included.

Then, we computed standard measures of sensitivity and specificity for diagnosis of current MDE with mixed features and (hypo)manic episode based on Bayesian principles, and computed receiver–operator curve (ROC) functions to evaluate the predictive value of the PARI/YARI score per subject [33]

All tests were two tailed. A *p*-value of 0.05 or less was considered indicative of significance. Analyses were conducted with Microsoft Office 365—Excel and IBM SPSS Statistics V26 software.

## 3. Results

### 3.1. Description of the Cohort

The sample was composed of 289 patients, of which 76.5% (221) were female. Mean age at evaluation was 15.1 ± 1.9 years; mean age at the onset of mood disorder symptoms was 11.9 ± 2.85 years (Table 1).

At clinical assessment, 207 (71.6% of the total sample) subjects had a current MDE, 41 of which had mixed features. Moreover, 14 subjects (4.8% of the total population sample) had a current episode characterized by cycling mood features and 27 subjects (9.3%) were diagnosed with an (hypo)manic episode (with or without mixed depressive features, Table 1).

MDD was the most common categorical diagnosis (65.4%, N = 189) and persistent depressive disorder with current intermittent MDE was diagnosed in 31 subjects (10.7% of the total sample). A bipolar or related disorder was diagnosed in 69 subjects (23.9%), of which 52 (18% of total population) were diagnosed with BD-NOS, 5 (1.7%) with BD type I, and 12 (4.2%) with BD type II (Table 1).

Current comorbidities were systematically assessed and at least one was identified in 57.4% (N = 166) of patients, with a minimum of 0 to a maximum of 4 DSM-5 comorbidities. Anxiety disorders were the most common comorbidity diagnosed in 22.2% (64) of patients. In terms of neurodevelopmental comorbidities, ADHD was diagnosed in 13.5% (N = 39) and learning disorders in 10% (N = 29). Other common comorbidities were eating disorders (8.7%, N = 25), PTSD (3.1%, N = 9), disruptive/impulse control disorders (2.4%, N = 7), and obsessive-compulsive/tic disorders (3.8%, N = 11, Table 1).

About a third of the sample (30.1%, N = 83) had severe depressive symptoms, as defined by a CDRS-R score of 55 or higher. Among all patients, KMRS had a threshold total score in 43 subjects (15.6%) and both CDRS-R and KMRS had a clinically significant score in 40 subjects (14.5% of the total sample).

Suicidal ideation was identified in 218 patients (75.4%) and 74 (25.6%) reported at least one suicidal attempt lifetime. Non-suicidal self-injury (NSSI, current or past) was identified in more than half of the sample (N = 165, 57.1%).

Forty-five subjects (15.6%) were hospitalized at least once in a psychiatric ward. Substance use was reported by 11.1% (32) of patients, but none of them reached threshold criteria for current substance use disorder.

Regarding the history of previous/concurrent treatment of the patients, when evaluated, there were no significant differences in each drug category and in psychotherapy between groups (Table 1).

### 3.2. Affective Reactivity Index, Parent- and Youth-Rated Modules

Mean ARI score was of 4.87 (range 0 to 12, SD 3.25) for parent-rated ARI modules (PARI) and of 5.88 (range 0 to 12, SD 3.18) for youth-rated ARI modules (YARI). PARI scores were significantly lower than YARI scores (*p* < 0.001, *t*-test 4.86). Chronbach’s alpha was of 0.839 for PARI and 0.806 for YARI.

We calculated the median value for both the PARI and YARI score and stratified the sample twice:

1. High versus low PARI score (median = 4.00);
2. High versus low YARI score (median = 6.00).

Inter-rater reliability for categorization of high versus low irritability between parents and patients was 0.32 (*p* < 0.001).

We studied in parallel demographic, clinical, and psychopathological features associated specifically with high parent-rated irritability (versus low parent-rated irritability) and with high self-rated irritability (versus low self-rated irritability) (Table 1, Table 2 and Table 3).

The two groups were similar in demographic features and in comorbidities, except for a higher rate of PTSD in patients with a PARI score above the median (5.70% vs. 0.70%, *p* = 0.017). When a Bonferroni correction for multiple comparisons is applied to comorbidities comparisons (adjusted α for the 7 comorbidities group studied = 0.007), this finding is no more significant.

Regarding mood disorder diagnosis, a PARI score > 4 was significantly more often associated with BD than UD diagnosis (30.5% vs. 17.6%, *p* = 0.01).

A PARI score > 4 was also more frequently associated with a current (hypo)manic episode (13.5% vs. 5.4%; *p* = 0.018) and mixed/cycling states (24.1% vs. 14.2%, *p* = 0.032), whereas a PARI score ≤ 4 was associated with current major depressive episodes (80.4% vs. 62.4%, *p* = 0.001). Patients with a YARI score higher than the cutoff of 6 were not significantly associated with differential incidence of any psychiatric diagnosis (Table 1).

### 3.3. ARI and Psychopathology, Bivariate Analyses

Analysis of (hypo)manic symptoms showed that both patients with a PARI score above the cutoff and patients with a YARI score above the cutoff are associated with higher KMRS total scores. Interestingly, patients with PARI total score above the cutoff had significantly higher scores in the KMSR items even if the irritability item had been removed (3.74 vs. 5.02, *p* = 0.014). This was reported also for patients with YARI total score above the cutoff (6.52 vs. 8.45, *p* = 0.001).

Analysis of single items, as expected, showed that both patients with PARI score above the cutoff and patients with YARI score above the cutoff have higher scores for the KMRS irritability item. Patients with a PARI score above the cutoff also have higher scores for euphoria, lability, increased goal directed activity, and reduced judice items. When a Bonferroni correction for multiple comparisons is applied to (hypo)manic symptoms (adjusted α for the 13 KMRS items studied = 0.0038), differences in euphoria and increased goal directed activities are no longer significant (Table 2).

Patients with a YARI score above the cutoff have a higher score for the lability item (Table 3), which is significant also after controlling for multiple comparisons.

Regarding depressive symptoms, no differences in the total CDRS-R score were found between patients with a PARI or YARI score above or below the median; however, as expected, patients with a YARI or PARI score above the median have higher scores for the irritability item. Patients with a PARI score above the median have higher score for the appetite item of CDRS-R and patients with a YARI score above the median have lower score for the difficulty in having fun item. When a Bonferroni correction for multiple comparisons is applied to depressive symptoms (adjusted α for the 17 CDRS-R items studied = 0.0029), differences in appetite disturbances are no longer significant (Table 2).

All other parent-rated tests (all CBCL scales, MASC-2 and CDI-2) have higher scores in patients with a PARI score above the median. Most scales of CBCL—notably internalizing, externalizing, total problems, and CBCL AAA Dysregulation Profile—are significantly higher in the groups with a YARI score above the cutoff, as also depressive (CDI-2) but not anxiety (MASC-2) self-rated symptoms. General functioning is significantly lower in patients with a PARI score higher than 4 (Table 2 and Table 3).

### 3.4. ARI and Psychopathology, Multivariate Analysis

We finally performed two multivariate logistic regression models to assess:

1. Factors significantly and independently associated with PARI > 4 (Table 4);
2. Factors significantly and independently associated with YARI > 6 (Table 5).

For both models, we tested as predictors variables found significantly different between groups at previous bivariate analyses, in more detail: diagnosis of bipolar disorder (present vs. absent); current episode (as an ordinal variable with three levels: non-mixed depression vs. mixed depression vs. hypo/mania); diagnosis of comorbid PTSD; KMRS score without irritability item, items on mood elation, lability, impaired judgement, and increase in goal directed activities; CDRS-R items on difficulties in having fun and appetite disturbance; CBCL internalizing, externalizing, and total problems scale scores; CBCL AAA (dysregulation) profile; CDI-2 self and parent module total score; MASC-2 parent module total score. Non-significant predictors were removed in a backward stepwise manner.

Both models are significant: chi square 70.2 and *p* < 0.001 for PARI model and chi square = 46.2 and *p* < 0.001 for model 2. R square is =0.33 for model 1 and =0.30 for YARI model.

Factors significantly and independently associated with PARI > 4 were: (a) more severe emotional dysregulation as measured with the CBCL AAA profile score, and (b) bipolar disorders diagnosis (Table 4).

Factors significantly and independently associated with YARI > 6 were: (a) lower CDRS-R difficulty of having fun item score, (b) greater CDI self-report score, (c) more severe emotional dysregulation as measured with the CBCL AAA profile score, and (d) greater CDRS-R appetite disturbance item score (Table 5).

## 4. Discussion

We identified significant differences in parent- and youth-rated modules of the affective reactivity index (PARI and YARI, respectively) significantly correlated with mood disorder diagnosis and distinctive psychopathological features. These findings add important evidence supporting the hypothesis that type and severity levels of irritability can help to diagnose type, severity, and clinical correlates of mood disorder episodes during juvenile ages. To our knowledge, this is the first report assessing diagnosis and psychopathological features associated with high versus low irritability score separately measured with the parent- and self-rated ARI.

The full range of ARI scores observed in this sample of subjects diagnosed with a major mood disorder varied from 0 to 12. Mean scores of both modules were meanly high (4.87 for PARI and 5.88 for YARI) and comparable to the ones reported in other studies on juvenile mood and behavioral disorders [10,31,34,35].

We found significant differences among clinical correlates of PARI and YARI scores. A PARI score above the median was significantly associated with BD versus MDD diagnosis, especially with BD-NOS (Table 1). This is not surprising, as it is well known that extreme irritability with temper outbursts may be the most common abnormal mood associated with PBD [36,37,38,39,40,41,42,43,44]. Also, a PARI score above the median was significantly associated with (hypo)manic and mixed episodes/cycling pattern. Indeed, around 70% of juvenile bipolar subjects are reported to present an ultra-ultra-rapid or ultra-rapid course pattern, whereas the classical episodic presentation of depressive and manic episodes is uncommon during juvenile ages [36,41,42].

A PARI score above the median was also significantly associated with KMRS total score and KMRS total score > 12, but not with the total CDRS-R depression score, confirming that parent-rated irritability is highly related to the presence and the severity of manic symptoms [45,46]. In particular, a PARI score above the median was strongly related to mood lability and poor judgment, but less with elation and increased goal directed activity, and not related to grandiosity (Table 2, see results section with Bonferroni corrections). Also, a PARI score above the median was significantly related with a lower global functioning as measured with C-GAS total score and greater parent rates of anxiety and depressive and emotional dysregulation problems as rated with MASC, CDI, and CBCL (Table 3). This is consistent with the picture of juveniles’ mixed states (or “dark manias”) characterized by extreme irritability, frequently appearing as explosive rages, and often associated with impulsive and reckless behaviors, violent gestures, and impulsive suicidal thoughts, threats, and behaviors [36,39,40,46].

Factors selectively and independently associated with a PARI score above the median in the multivariable logistic regression model were bipolar disorder diagnosis and CBCL AAA severe dysregulation profile, confirming that extreme irritability is strictly related to pediatric mania, and also that a CBCL AAA total score above 210 can be highly indicative of a pediatric BD diagnosis [47].

On the other hand, youth-rated irritability above the median (YARI > 6) was significantly and tautologically associated with greater levels of irritability measured with both the irritability items of the CDRS-R and KMRS, but did not correlate with the severity of the other manic symptoms except for mood lability (Table 2). Patients with a high YARI score appear to constitute a depressive phenotype with less anhedonia and apathy, but not with lower overall severity, characterized in particular by appetite/food-intake dysregulation and mood lability.

Limitations of this study include that ARI was developed to specifically assess chronic irritability in subjects with SMD/DMDD. A more complete measure of irritability should be developed to assess episodic irritability in patients with major mood disorders. Referral bias for great severity of referred patients and the lack of longitudinal follow up are other relevant limitations. Also, lack of demographic and clinical information about parents’ sample did not allow including such information as covariates in the present study.

## 5. Conclusions

In this study, we identified significant differences in parent- and youth-rated modules of the affective reactivity index significantly correlated with mood disorder diagnosis and distinctive psychopathological features.

Factors significantly and independently associated with high versus low parent-rated ARI score were: more severe emotional dysregulation and bipolar disorders diagnosis. Factors significantly and independently associated with high versus low self-rated ARI score were: lower CDRS-R difficulty of having fun item score, greater CDI-2 self-report score, more severe emotional dysregulation, and greater CDRS-R appetite disturbance item score. High parent-rated irritability was strictly related with a bipolar disorder diagnosis, whereas high youth-rated irritability was related to a depressive phenotype characterized by appetite/food-intake dysregulation, mood lability, and less anhedonia and apathy.

The present report provides researchers and clinicians with important evidence supporting the hypothesis that type and severity levels of irritability can help to diagnose type, severity, and clinical correlates of mood disorder episodes during juvenile ages.

## Figures and Tables

**Table 1:** Descriptive characteristics of study patients.

		PARI (Parent-Report ARI)			YARI (Self-Report ARI)		
Measure	All Subjects	Score ≤ 4	Score > 4	*p*-Value (Statistic)	Score ≤ 6	Score > 6	*p*-Value (Statistic)
Subjects (N)	289	148	141	/	135	154	/
Female sex (%)	76.5	74.3	78.7	0.378	76.3	76.6	0.948 (0.004)
Ages, years (mean ± SD)							
At first major affective episode	11.9 ± 2.85	12.1 ± 2.64	11.6 ± 3.05	0.177 (1.35)	11.9 ± 2.61	11.7 ± 3.06	0.66 (0.44)
At evaluation	15.1 ± 1.90	15.0 ± 1.75	15.1 ± 2.04	0.939 (0.08)	15.0 ± 1.86	15.1 ± 1.93	0.665 (0.43)
Diagnosis (%, N)							
Bipolar Disorders	23.9 (69)	17.6 (26)	30.5 (43)	0.01 (6.64)	21.5 (29)	26.0 (40)	0.371 (0.799)
BD Type I or II	5.90 (17)	4.10 (6)	7.80 (11)	0.176 (1.83)	4.40 (6)	7.10 (11)	0.331 (0.946)
BD NOS	18.0 (52)	13.5 (20)	22.7 (32)	0.042 (4.13)	17.0 (23)	18.8 (29)	0.692 (0.157)
Unipolar Disorders	76.1 (220)	82.4 (122)	69.5 (98)	0.01 (6.64)	78.5 (106)	74.0 (114)	0.371 (0.799)
Persistent Depressive Disorder	10.7 (31)	12.8 (19)	8.50 (12)	0.235(1.41)	11.9 (16)	9.70 (15)	0.563 (0.335)
Major Depressive Disorder	65.4 (189)	69.6 (103)	61.0 (86)	0.124 (1.36)	66.7 (90)	64.3 (99)	0.671 (0.180)
Current mood Episode (%, N)							
Major depressive	71.6 (207)	80.4 (119)	62.4 (88)	0.001(11.5)	74.8 (101)	68.8 (106)	0.260 (1.26)
Mixed/cycling	19.0 (55)	14.2 (21)	24.1 (34)	0.032(4.62)	17.8 (24)	20.1 (31)	0.611 (0.258)
Hypo(manic)	9.30 (27)	5.40 (8)	13.5 (19)	0.018(5.55)	7.40 (10)	11.0 (17)	0.290 (1.12)
Psychiatric co-morbidities (%, N)							
Any comorbidity	57.4 (166)	57.4 (85)	57.4 (81)	0.998 (0.00)	56.3 (76)	58.4 (90)	0.713 (0.135)
ADHD	13.5 (39)	11.5 (17)	15.6 (22)	0.306(1.05)	13.3 (18)	13.6 (21)	0.940 (0.006)
Anxiety	22.2 (64)	23.1 (34)	21.3 (30)	0.705(0.143)	26.9 (36)	18.2 (28)	0.077 (3.126)
Disruptive/Impulse dyscontrol	2.40 (7)	2.70 (4)	2.10 (3)	0.751(0.101)	2.20 (3)	2.60 (4)	0.999 (0.043)
OCD/tic Disorders	3.80 (11)	4.10 (6)	3.50 (5)	0.822(0.051)	4.40 (6)	3.20 (5)	0.595 (0.282)
Eating disorders	8.70 (25)	9.50 (14)	7.80 (11)	0.616(0.251)	5.90 (8)	11.0 (17)	0.123 (2.380)
Learning disorder	10.0 (29)	9.50 (14)	10.6 (15)	0.739(0.111)	8.90 (12)	11.0 (17)	0.544 (0.368)
PTSD	3.10 (9)	0.70 (1)	5.70 (8)	0.017(5.98)	2.20 (3)	3.90 (6)	0.414 (0.668)
Substance abuse (ever)	11.1 (32)	8.10 (12)	14.2 (32)	0.100 (2.71)	8.10 (11)	13.6 (21)	0.138 (2.201)
Self-harm status (mean ± SD or %, N)							
C-SSRS screening	1.85 ± 1.55	1.70 ± 1.50	2.02 ± 1.59	0.088 (1.71)	1.76 ± 1.56	1.93 ± 1.53	0.393 (−0.86)
Suicidal Ideation, lifetime	75.4 (218)	75.0 (111)	75.9 (107)	0.861(0.031)	74.1 (100)	76.6 (118)	0.615 (0.252)
Suicide attempters, lifetime	25.6 (74)	24.3 (36)	27.0 (38)	0.609(0.261)	23.7 (32)	27.3 (42)	0.488 (0.481)
NSSI, current or past	57.1 (165)	54.1 (80)	60.3 (85)	0.285(1.14)	53.3 (72)	60.4 (93)	0.227 (1.46)
Psychiatric Hospitalization (%, N)	15.6 (45)	18.9 (28)	12.1 (17)	0.108 (2.58)	16.3 (22)	14.9 (23)	0.750 (0.101)
Medications (%, N)							
Antidepressants	14.9 (43)	1.42 (21)	15.6 (22)	0.736(0.114)	16.3 (22)	13.6 (21)	0.526 (0.402)
First-generation antipsychotics	1.40 (4)	2.00 (3)	0.70 (1)	0.623(0.919)	2.20 (3)	0.60 (1)	0.343 (1.30)
Second-generation antipsychotics	30.4 (88)	34.5 (51)	26.2 (37)	0.129(2.30)	25.9 (35)	34.4 (53)	0.118 (2.45)
Benzodiazepines	18.3 (53)	18.2 (27)	18.4 (26)	0.966(0.002)	15.6 (21)	20.8 (32)	0.999 (0.043)
Antiepileptics	6.60 (19)	4.70 (6)	3.50 (12)	0.195(1.681)	4.40 (6)	3.20 (5)	0.252 (1.31)
Lithium	4.80 (14)	2.80 (8)	2.10 (6)	0.649(0.207)	5.20 (8)	4.50 (7)	0.800 (0.064)
Psychotherapy (%, N)	72.6 (201)	75.5 (108)	69.4 (93)	0.254 (1.30)	72.3 (94)	72.8 (107)	0.929 (0.008)

Statistics: Unpaired *t*-test for continuous and χ^2^ for categorical measures. Abbreviations: Bipolar Disorder (BD), BD not otherwise specified (BD-NOS), obsessive-compulsive disorder (OCD), post-traumatic stress disorder (PTSD), Columbia suicide severity rating scale (C-SSRS), non-suicidal self-injury (NSSI).

**Table 2:** Clinician-Rated Psychopathological features versus Self- and Parent-Rated ARI scores.

		PARI (Parent-Report ARI)			YARI (Self-Report ARI)		
Measure	All Subjects	Score ≤ 4	Score> 4	*p*-Value (Statistic)	Score ≤ 6	Score> 6	*p*-Value (Statistic)
Clinician rated Depressive features (CDRS-R)							
CDRS-R total score (mean ± SD)	48.4 ± 12.2	47.3 ± 11.4	49.5 ± 13.0	0.125 (−1.54)	48.5 ± 11.9	48.3 ± 12.4	0.853 (0.180)
Severe depression (CDRS-R > 55) (%,N)	30.1 (83)	25.4 (36)	35.1 (47)	0.078 (3.099)	31.8 (41)	28.6 (42)	0.562 (0.337)
Irritability item score (mean ± SD)	3.47 ± 1.39	2.89 ± 1.16	4.08 ± 1.35	<0.001 (−7.78)	2.96 ± 1.25	3.90 ± 1.35	<0.001 (−5.89)
Difficulties in having fun (mean ± SD)	3.54 ± 1.55	3.55 ± 1.55	3.53 ± 1.55	0.917 (0.105)	3.88 ± 1.64	3.26 ± 1.41	0.001 (3.325)
Appetite Disturbances (mean ± SD)	2.61 ± 1.28	2.43 ± 1.36	2.8 ± 1.17	0.018 (−2.39)	2.44 ± 1.28	2.74 ± 1.27	0.055 (−1.93)
Clinician rated Manic features (K-MRS)							
KMRS total (mean ± SD)	7.55 ± 4.93	6.25 ± 3.77	8.95 ± 5.61	<0.001 (−4.63)	6.52 ± 4.4	8.45 ± 5.19	0.001 (−3.29)
KMRS > 12 (%,N)	14.80 (43)	9.10 (13)	22.7 (30)	0.002 (9.676)	14.1 (18)	17% (25)	0.503 (0.45)
KMRS > 12 and CDRS−R > 29 (%,N)	14.50 (40)	7.70 (11)	22.0 (29)	0.001 (11.26)	12.5 (16)	16.3 (24)	0.369 (0.806)
KMRS total (minus irritability) (mean ± SD)	4.35 ± 4.14	3.74 ± 3.34	5.02 ± 4.79	0.014 (−2.49)	3.88 ± 3.40	4.77 ± 4.65	0.082 (−1.74)
Elation and expansive mood (mean ± SD)	1.30 ± 0.62	1.22 ± 0.52	1.38 ± 0.70	0.034 (2.13)	1.22 ± 0.56	1.36 ± 0.67	0.068 (−1.83)
Irritability and anger (mean ± SD)	2.56 ± 0.92	2.2 ± 0.85	2.94 ± 0.84	<0.001 (−7.09)	2.28 ± 0.92	2.80 ± 0.86	<0.001 (−4.75)
Mood lability item score (mean ± SD)	2.64 ± 0.94	2.41 ± 0.94	2.90 ± 0.88	<0.001 (−4.33)	2.35 ± 0.95	2.89 ± 0.87	<0.001 (−4.84)
Increased goal directed activities (mean ± SD)	1.17 ± 0.52	1.09 ± 0.38	1.26 ± 0.63	0.015 (−2.46)	1.11 ± 0.44	1.23 ± 0.58	0.06 (−1.89)
Poor judgment (mean ± SD)	1.24 ± 0.61	1.12 ± 0.40	1.38 ± 0.76	0.001 (−3.41)	1.19 ± 0.54	1.28 ± 0.67	0.21 (−1.26)
Global Functioning (C-GAS)							
C-GAS (mean ± SD)	51.6 ± 7.43	53.6 ± 7.63	49.6 ± 6.66	<0.001 (4.55)	52.0 ± 7.64	51.2 ± 7.25	0.388 (0.87)

Statistics: Unpaired *t*-test for continuous and χ^2^ for categorical measures. Abbreviations: Children depression rating scale (CDRS-R), Kiddie-mania rating scale (KMRS), Clinical Global Assessment Scale (C-GAS).

**Table 3:** Self- or Parent-Rated psychopathological features versus Self and Parent-Rated ARI scores.

		PARI (Parent-Report ARI)			YARI (Self-Report ARI)		
Measure	All Subjects	Score ≤ 4	Score > 4	*p*-Value (Statistic)	Score ≤ 6	Score > 6	*p*-Value (Statistic)
Self-rated symptoms (MASC-2, CDI-2)							
Anxiety (MASC-2) (mean ± SD)	66.4 ± 14.1	65.8 ± 14.7	66.9 ± 13.4	0.569 (−0.57)	64.6 ± 15.2	67.9 ± 12.9	0.083 (−1.74)
Depression (CDI-2) (mean ± SD)	72.1 ± 13.2	71.5 ± 13.1	72.7 ± 13.2	0.47 (−0.72)	68.5 ± 13.4	75.3 ± 12.2	<0.001 (−3.95)
Parent rated symptoms (MASC-2, CDI-2, CBCL)							
Anxiety (MASC-2) (mean ± SD)	68.5 ± 13.5	65.3 ± 13.3	71.8 ± 13.1	<0.001 (−3.61)	66.7 ± 14.5	70.2 ± 12.5	0.055 (−1.93)
Depression (CDI-2) (mean ± SD)	67.4 ± 10.3	64.0 ± 8.92	70.8 ± 10.4	<0.001 (−5.18)	65.5 ± 9.72	69.1 ± 10.5	0.009 (−2.65)
CBCL internalizing (mean ± SD)	73.7 ± 6.88	71.9 ± 7.13	75.6 ± 6.03	<0.001 (−4.37)	72.6 ± 7.33	74.7 ± 6.27	0.014 (−2.47)
CBCL externalizing (mean ± SD)	61.7 ± 9.01	58.0 ± 7.61	65.8 ± 8.71	<0.001 (−7.46)	60.2 ± 8.75	63.1 ± 9.07	0.011 (−2.55)
CBCL total (mean ± SD)	68.7 ± 6.37	66.0 ± 6.17	71.8 ± 5.09	<0.001 (−7.88)	67.1 ± 6.74	70.2 ± 5.64	<0.001 (−3.80)
CBCL AAA (mean ± SD)	201 ± 21.8	192 ± 18.3	213 ± 20.0	<0.001 (−8.67)	197 ± 21.7	206 ± 20.9	0.001 (−3.43)
CBCL DESR (180 < CBCL AAA ≤ 210) (%,N)	47.5 (116)	53.1 (69)	41.2 (47)	0.064 (3.42)	50.4 (60)	44.8 (56)	0.380 (0.772)
CBCL Severe Dysregulation (CBCL AAA > 210) (%,N)	35.2 (86)	18.5 (24)	54.4 (62)	<0.001 (34.4)	26.1 (31)	44.0 (55)	0.003 (8.61)

Statistics: Unpaired *t*-test for continuous and Chi^2^ for categorical measures. Abbreviations: Children Depression Inventory (CDI), Multidimensional Anxiety Scale for Children (MASC), Child Behavior Checklist (CBCL), Deficient Emotional Self-Regulation (DESR), CBCL AAA (sum of the score con attention problems + aggression + anxious-depressed syndromic scales of CBCL).

**Table 4:** Multivariate logistic regression model of factors associated with PARI > 4.

Factors	Odds Ratio [95% CI]	Wald	*p*-Value
CBCL AAA profile	1.06 [1.04–1.08]	44.6	<0.0001
Bipolar Disorder diagnosis	2.14 [1.06–4.32]	4.51	0.034

**Table 5:** Multivariate logistic regression model of factors associated with YARI > 6.

Factors	Odds Ratio [95% CI]	Wald	*p*-Value
Lower CDRS-R difficulties in having fun	0.533 [0.409–0.694]	21.9	<0.0001
CDI-2 self-rated	1.05 [1.02–1.04]	11.7	0.001
CBCL AAA	1.03 [1.01–1.09]	9.02	0.003
CDRS-R appetite disturbance	1.46 [1.09–1.96]	6.27	0.012

Statistics: Logistic regression. Predicted variable is high PARI score, according to the cutoff of 4, in model 1 and high YARI score, according to the cutoff of 6, in model 2. We tested as predictors variables found significantly different between groups at previous bivariate analyses, in more detail: Diagnosis of bipolar disorder (present vs absent), current episode (as an ordinal variable with three levels: Non mixed depression VS mixed depression VS hypo/mania), diagnosis of comorbid PTSD, KMRS score without irritability item), KMRS items on mood elation, lability, impaired judgement, and increase in goal directed activities; CDRS-R items on difficulties in having fun and appetite disturbance, CBCL internalizing, externalizing and total problems scale scores, CBCL AAA (dysregulation) profile, CDI-2 self and parent module total score, MASC-2 parent module total score. Nonsignificant predictors were removed in a backward stepwise manner the predictors which were significant in the final model are presented with their Odds Ratio (exp B) with 95% confidence intervals, wald test score and *p* value. Intercept is included in the models. Both models are significant: chi square 70.2 and *p* < 0.001 for model 1 and chi square = 46.2 and *p* < 0.001 for model 2. R square is =0.33 for model 1 and =0.30 for model 2. Abbreviations: Children depression rating scale (CDRS-R), Children Depression Inventory (CDI), Child Behavior Checklist (CBCL), CBCL AAA (sum of the score con attention problems + aggression + anxious-depressed syndromic scales of CBCL).

## Data Availability

The data presented in this study are available on request from the corresponding authors. The data are not publicly available because of ethical concerns.
